# Prospective Clinical and Pharmacological Evaluation of the Delcath System’s Second-Generation (GEN2) Hemofiltration System in Patients Undergoing Percutaneous Hepatic Perfusion with Melphalan

**DOI:** 10.1007/s00270-017-1630-4

**Published:** 2017-04-27

**Authors:** Eleonora M. de Leede, Mark C. Burgmans, T. Susanna Meijer, Christian H. Martini, Fred G. J. Tijl, Jaap Vuyk, Arian R. van Erkel, Cornelis J. H. van der Velde, Ellen Kapiteijn, Alexander L. Vahrmeijer

**Affiliations:** 1 Department of Surgery, Leiden University Medical Centre, Albinusdreef 2, 2300 RC Leiden, The Netherlands; 2 Department of Radiology, Leiden University Medical Centre, Albinusdreef 2, 2300 RC Leiden, The Netherlands; 3 Department of Anesthesiology, Leiden University Medical Centre, Albinusdreef 2, 2300 RC Leiden, The Netherlands; 4 Department of Cardio-Thoracic Surgery, Leiden University Medical Centre, Albinusdreef 2, 2300 RC Leiden, The Netherlands; 5 Department of Medical Oncology, Leiden University Medical Centre, Albinusdreef 2, 2300 RC Leiden, The Netherlands

**Keywords:** Cancer, Liver, Uveal melanoma, Colorectal cancer, Interventional oncology, Chemoembolisation

## Abstract

**Introduction:**

Percutaneous hepatic perfusion (PHP) with melphalan is an effective treatment for patients with hepatic metastases, but associated with high rates of bone marrow depression. To reduce systemic toxicity, improvements have been made to the filtration system. In pre-clinical studies, the Delcath System’s GEN2 filter was superior to the first-generation filters. In this clinical study, we analysed the pharmacokinetics and toxicity of PHP using the new GEN2 filter.

**Methods and Materials:**

Starting February 2014, two prospective phase II studies were initiated in patients with hepatic metastases from ocular melanoma or colorectal cancer. In 10 PHP procedures performed in the first 7 enrolled patients, blood samples were obtained to determine filter efficiency and systemic drug exposure. PHP was performed with melphalan 3 mg/kg with a maximum of 220 mg. Complications were assessed according to CTCAE v4.03. Response was assessed according to RECIST 1.1.

**Results:**

Pharmacokinetic analysis of blood samples showed an overall filter efficiency of 86% (range 71.1–95.5%). The mean filter efficiency decreased from 95.4% 10 min after the start of melphalan infusion to 77.5% at the end of the procedure (*p* = 0.051). Bone marrow depression was seen after up to 80.0% of 10 procedures, but was self-limiting and mostly asymptomatic. No hypotension-related complications or procedure-related mortality occurred.

**Conclusion:**

The GEN2 filter has a higher melphalan filter efficiency compared to the first-generation filters and a more consistent performance. PHP with the GEN2 filter appears to have an acceptable safety profile, but this needs further validation in larger studies.

## Introduction

Percutaneous hepatic perfusion (PHP) is an innovative, minimally invasive procedure that is gaining interest as a therapeutic option for patients with hepatic malignancies. A recently published randomised controlled trial (RCT) has shown superiority of PHP over best alternative care in patients with hepatic metastases from ocular and cutaneous melanoma [1]. Furthermore, small prospective cohort studies have shown promising results in patients with secondary liver tumours as well as primary liver tumours [2–7]. Wide acceptance of PHP in clinical practice has been halted due to concerns about the safety profile of PHP. The most notable complication of PHP is bone marrow depression resulting in anaemia, neutropenia, and/or thrombocytopenia. Reported rates of complications related to bone marrow depression vary from 43.7 to 85.7% [8]. In PHP, the liver vasculature is isolated from the systemic circulation using percutaneously inserted catheters. A microcatheter is placed in the hepatic artery to deliver a high dose of the chemotherapeutic agent melphalan. Prior to the start of infusion of the chemotherapeutic drug, a double-balloon catheter is placed in the inferior caval vein (ICV). The balloons prevent leakage of chemotherapeutics to the systemic circulation by occluding the ICV at the level of the atriocaval junction and infra-hepatic ICV. Through catheter side holes located in between the two balloons, the chemosaturated blood returning through the hepatic veins is aspirated and the blood is then pumped through an extracorporeal filtration system. After filtration, the blood is returned to the patient through a catheter in the internal jugular vein [8]. The high rate of bone marrow depression associated with PHP indicates that systemic exposure to chemotherapeutic drugs does occur. This may result from failure to achieve complete isolation of the liver vasculature or from incomplete extraction of chemotherapeutics by the hemofiltration system. In a phase I trial including 28 patients treated with PHP, pharmacological analyses of blood samples demonstrated a mean filter extraction rate of 77% (range 58.2–94.7%) [9]. In this study, and most of the other published studies, PHP was performed using a first-generation hemofiltration system. In 2012, a second-generation detoxification cartridge (GEN 2 filter; Delcath Systems, New York, NY, USA) was made commercially available. Compared to the first-generation hemofiltration system, the GEN 2 filter has been modified in several ways to improve the filter extraction rate. The activated carbon particles have been changed in shape (from granular to spherical), density (from 0.600 to 0.560 to 0.195–0.185 g/ml), size (mean ± standard deviation from 1363 ± 457 to 720 ± 102 μm), and volume per cartridge (from 500 to 550 ml). In a porcine study, the extraction rate of the GEN 2 filter was 99 ± 0.4% [10]. Initial clinical experiences seem to indicate that the use of the GEN 2 filter may indeed reduce systemic toxicity [7]. In 2014, we initiated two phase II trials investigating PHP with the GEN 2 filter in patients with hepatic metastases from either ocular melanoma or colorectal carcinoma. As part of these trials, we obtained blood samples in a subset of patients to investigate the pharmacokinetics of PHP with the GEN 2 filter. Our hypothesis was that the use of the GEN 2 filter would result in a higher filter extraction rate and lower incidence of bone marrow depression compared to those reported after PHP with the first-generation filter. The primary objective of this pharmacological study was to determine the melphalan filter efficiency of the GEN 2 filter. The objective of the phase II studies was to analyse the safety and efficacy of PHP with melphalan.

## Materials and Methods

### Study Design and Patients

Patients were included in one of two prospective phase II studies on PHP with melphalan, starting February 2014. In this pharmacological study, the first consecutive seven patients treated with PHP were included as part of the aforementioned phase II studies. In the first three patients, pharmacological samples were also obtained during the second PHP procedure.

Thus, pharmacological data of 10 PHP procedures in 7 patients were analysed. The phase II studies and the presented pharmacological study were approved by the Local Medical Ethics Committee of the Leiden University Medical Centre. Patients were potential candidates for one of the two phase II studies, if they had histologically proven, unresectable metastases confound to the liver from either ocular melanoma or colorectal carcinoma. Patients were ineligible for surgical resection because of diffusely spread of liver disease or a metastasis not accessible for surgical resection or radiofrequency ablation, as evaluated by a multidisciplinary liver team of hepatic surgeons, medical oncologists, and interventional radiologists. Both phase II studies had similar inclusion criteria: life expectancy >4 months, resection of the primary tumour >4 weeks prior to PHP, aspartate aminotransferase (AST), alanine aminotransferase (ALT), and alkaline phosphatase ≤5 times upper limit of normal, leucocyte count ≥3.0 × 10^9^/l, platelet count ≥100 × 10^9^/l, and estimated GFR ≥40 ml/min. Exclusion criteria were a World Health Organisation (WHO) performance status of ≥2, age <18 and >65 years, less than 40% healthy liver tissue based on computed tomography (CT) or magnetic resonance imaging (MRI), evidence of extrahepatic disease or coagulation disorders: activated partial thromboplastin time (APTT) >32.5 s and prothrombin time (PT) >13.7 s. Contrast-enhanced CT of chest, abdomen (arterial and venous phase), and brain was performed to exclude extrahepatic disease and detect vascular variants precluding PHP. All patients underwent pre-procedural angiography with cone beam CT. The later was used to exclude extrahepatic enhancement and vascular tumour supply from extrahepatic collaterals. All patients provided written informed consent for the study. Patients were routinely scheduled to undergo two PHP procedures with a six-week interval, in case there was no progression of disease after the first PHP.

### PHP Procedure

**Fig. 1:**
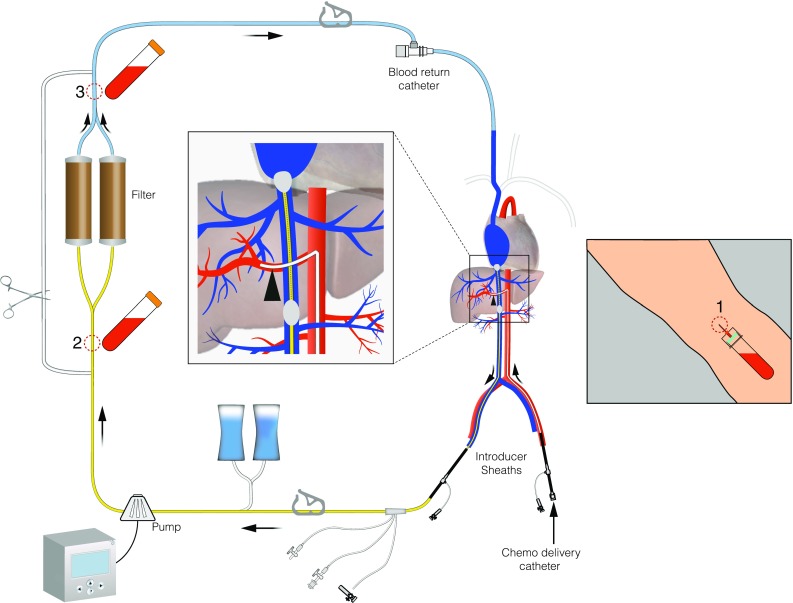
Schematic overview of PHP circuit. Indicated are the pharmacokinetic sampling points

Details of the PHP procedure have been described previously [8]. The following description is a summary of the most relevant parts of the procedure. Procedures were performed under general anaesthesia in the angiography room by a dedicated team of an interventional radiologist, anaesthesiologist, and perfusionist. After creation of vascular accesses to both internal jugular veins, the right common femoral vein, and left hepatic artery, heparin was administrated to achieve an activated clotting time (ACT) of >400 s. A 2.7F microcatheter (Progreat, Terumo, Tokyo, Japan) was placed in the hepatic artery to deliver melphalan. A double-balloon catheter (Isofuse Isolation Aspiration catheter, Delcath Systems Inc., New York, USA) was positioned in the ICV, and the balloons were inflated to prevent the flow of chemosaturated blood to the systemic circulation (Fig. 1). During set-up and initiation of the extracorporeal filtration circuit, sufficient blood pressure was maintained by the anaesthesiologist by administration of fluids and intravenous infusion of norepinephrine and/or phenylephrine. All PHP procedures were performed with the GEN 2 filtration system. Melphalan (Alkeran, Aspen, Dublin, Ireland) was infused at a dose of 3 mg/kg (with a maximum of 220 mg) at a rate of 0.4 ml/s in about 30 min. After melphalan infusion, extracorporeal circulation of blood returning through the hepatic veins was maintained for an additional 30 min (‘washout’ period). At the end of the procedure, protamine sulphate was administrated to reverse the effects of heparin. Patients were monitored in a medium or intensive care unit 12–24 h after the procedure. Patients were discharged from the hospital at day 3 after PHP.

### Pharmacokinetic Sampling

Blood samples were taken simultaneously from the median cubital vein as well as of the tubing before and after the filter of the extracorporeal system starting 10 min after commencement of melphalan infusion (T_10_), at the end of melphalan infusion (*T*_end infusion_) and at the end of the washout period (*T*_end washout)_ (see Fig. 1). In addition to this, venous (systemic) blood samples were obtained 10 and 20 min after the start of the washout period, at the end of the washout period, and 5, 30, 60, and 120 min after the end of the washout period. Blood was drawn in 10-ml sodium heparin tubes and placed in ice immediately after collection. Directly after the PHP procedure, the blood samples were centrifuged for 10 min at 1000×*g* at room temperature. After centrifugation, the plasma was split into two aliquots and stored in cryovials at −70 °C until analysis. All samples were analysed for melphalan by a high-performance liquid chromatographic analysis with ultraviolet detection as previously described [11]. The detection limit of melphalan in plasma was 0.5 µg/ml. The intra-assay coefficients of variation were 2.5% for melphalan in plasma in the concentration range of 0.5–5.0 µg/ml, and the inter-assay coefficients of variation were 12.4% for melphalan in plasma in the concentration range of 0.5 µg/ml, and 3.6% for melphalan in plasma in the concentration range of 5.0 µg/ml.

### Safety and Efficacy of PHP

Blood tests were performed on each patient prior to treatment, on days 1, 2, 3, 9, 12, 15, and 18 after PHP and then weekly, until both blood cell count and liver function tests were normalised or reduced to grade I–II toxicity according to the common terminology criteria for adverse events v4.03 (CTCAE v4.03). Routine study blood tests included full blood count, APTT, PT, international normalised ratio (INR), glucose, creatinine, sodium, potassium, bilirubin, amylase, alkaline phosphatase, ALT, AST, lactate dehydrogenase (LDH), γ-glutamyl transferase, protein, albumin, bicarbonate. Routine follow-up included visits to the outpatient clinic at 1 and 6 weeks and then every three months as well as telephonic consultation at days 9, 12, 15, and 18. Patients underwent CECT of the abdomen and chest (including arterial phase of the liver) 4 and 12 weeks after the first PHP procedure and every 3 months thereafter. In patients with poor visibility of metastases on CT, multiphase MRI of the liver was performed instead of CECT of the abdomen. If the CECT at 4 weeks post-PHP did not demonstrate disease progression and no complications occurred during the first PHP that contra-indicated repeated treatment, patients underwent a second PHP procedure as per protocol.

### Outcome Assessment

Technical success was defined as the successful delivery of the prescribed dose of melphalan. The mean filter efficiency of the GEN 2 filters was determined by calculation of the difference between the areas under the plasma melphalan concentration–time curves (AUC) before and after the filter. The AUCs were calculated with the trapezoidal rule. The overall mean filter efficiency was calculated as follows: [(pre-filter AUC) − (post-filter AUC)/(pre-filter AUC)] × 100. For the filter efficiency at a specific time point, the filter efficiency was calculated using the pre- and post-filter concentrations [(pre-filter concentration *T*_x_) − (post-filter concentration *T*_x_)/(pre-filter concentration *T*_x_)] × 100. The maximum concentration (*C*_max_) was defined as the peak systemic concentration of melphalan during a PHP procedure. Post-procedural blood test abnormalities, toxicity, and adverse events were assessed according to CTCAE v4.03. Haematological laboratory disorders occurring within 3 days after PHP were categorised as ‘early’ and those occurring more than 3 days after PHP as ‘late’. Early haematological complications were considered to be related to the procedure itself, i.e. to the dilution of blood as a result of fluid administration and/or to haemolysis by the hemofiltration system. Late haematological complications were most likely attributable to bone marrow depression as a result of melphalan toxicity. CT and MRI scans were assessed by an independent abdominal radiologist according to Response Evaluation Criteria for Solid Tumours version 1.1 (RECIST 1.1). Time to progression and overall survival were assessed.

### Statistical Analysis

The filter extraction rates for all perfusions at different time points are expressed as mean ± standard deviation (SD). The mean filter efficiency rates and mean melphalan plasma concentration were compared using a paired *t* test. Time to progression and overall survival were expressed in months as mean and median ± SD. All data were analysed using SPSS software for Windows version 20 (SPSS, Chicago, IL, USA). Graphs were created using GraphPad Prism 6 Software for Windows (GraphPad Software, La Jolla, CA, USA). A difference was considered significant when *p* < 0.05.

## Results

### Patients and Procedure

Patients and tumour characteristics of the 7 patients are listed in Table 1. Median age at the time of treatment was 57 years (range 42–64 years); 5 patients were males. All patients received previous treatment for their hepatic metastases, such as systemic chemotherapy, radiofrequency ablation (RFA), or immunotherapy in a clinical trial. Four out of the seven patients underwent two technically successful PHP procedures as per protocol; however, not all these procedures were included in this pharmacological study. Three patients underwent only one PHP procedure. One patient was reluctant to undergo a second PHP as the first procedure was complicated by pancytopenia with severe bacterial pharyngitis. In another patient, the CT 6 weeks after the first procedure showed progression of colorectal hepatic metastases and this patient did therefore not undergo a second PHP procedure. The third patient developed a pulmonary embolus three weeks after the procedure and was reluctant to undergo a second PHP. All ten PHP procedures were technically successful. Median duration of infusion for all procedures was 45 min (range 39–55 min). The overall mean duration of the entire PHP procedure was 4:02 h (range 3:26–4:45 h). The perfusion parameters are listed in Table 2. All patients were successfully treated with the planned dose of 3 mg/kg body weight, with a maximum dose of 220 mg of melphalan. The median follow-up was 24 months (interquartile range 9.0–26.5 months).

**Table 1:** Characteristics of 7 patients with unresectable liver metastases treated with percutaneous hepatic perfusion

PT	Sexe/age	Type of cancer	Time between first diagnosis and PHP (months)	Time between diagnosis liver metastases and PHP (months)	No. PHP’s	Best response	Time to progr. (months)	Location of progression	Status	Follow-up after first perfusion (months)
1	M, 57	UM	105	34	2	PR	28	Liver	Alive	28^a^
2	F, 62	UM	36	6	1	PR	9	Liver	Dead	11
3	M, 42	UM	36	3	1	PR	11	Bone, liver	Alive	26
4	M, 58	CRC	34	34	1	SD	1	Lymph node, LTR	Dead	7
5	M, 46	CRC	28	27	2	PR	5	Lung	Alive	27
6	F, 43	UM	40	16	2	PR	14	Liver	Alive	25^b^
7	M, 64	CRC	30	30	2	SD	5	Lung	Alive	24

*PT* patient, *PHP* percutaneous hepatic perfusion, *UM* uveal melanoma, *CRC* colorectal cancer, *LTR* local tumour recurrence at colonic anastomosis

^a^2nd perfusion was followed by radiofrequency ablation (RFA) of 6 small residual tumours

^b^2nd perfusion was followed by RFA of 3 small residual tumours. Because of hepatic progression, another 2 perfusions were performed

**Table 2:** Treatment parameters for the ten procedures

Procedure	Dose melphalan (mg)	Duration PHP procedure (h)	Duration of melphalan infusion (min)	Duration of filtration (min)	Location of infusion
1	220	3:58	NR	75	PHA
2	180	3:26	51	88	RHA (144 mg) and LHA (36 mg)
3	220	3:05	50	85	PHA
4	220	3:28	40	81	LHA (180 mg) and RHA (40 mg)
5	165	3:54	40	82	PHA
6	210	3:59	39	98	PHA
7	220	4:44	43	79	RHA (110 mg) and LHA (110 mg)
8	220	4:45	45	80	PHA (110 mg) and RHA (110 mg)
9	210	3:55	40	95	CHA
10	220	4:15	55	84	PHA (110 mg) and replaced RHA(110 mg)

*NR* not recorded, *PHA* proper hepatic artery, *RHA* right hepatic artery, *LHA* left hepatic artery, *CHA* common hepatic artery

### Pharmacokinetic Analysis

**Fig. 2:**
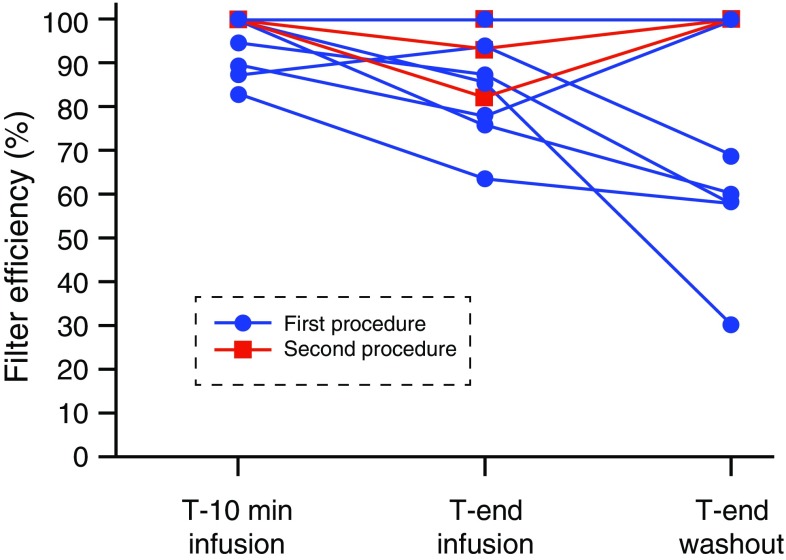
Filter efficiency per patient at different time points during the procedure. The mean filter efficiency was calculated at three time points during the 10 procedures. First at ten minutes after the start of the melphalan infusion, then at the end of the melphalan infusion, and at the end of the washout period

Heparinised blood samples were successfully obtained during all ten PHP procedures as per protocol. A summary of the *C*_max_, area under the curve (AUC), and filter efficiency is shown in Table 3. The overall mean filter efficiency during 10 PHP procedures was 86.0% (range 71.1–95.5%). No significant differences were observed in filter efficiency and systemic concentrations between the first procedure and second procedure in the patients who underwent two procedures. Figure 2 illustrates the changes in filter efficiency during the 10 PHP procedures. The mean filter efficiency at specific time points decreased from 95.4% (range 82.7–100%) at *T*_10_ to 77.5% (range 30–100%) at *T*_end washout_ (*p* = 0.051).

**Table 3:** Outcomes of filter efficiency in 10 procedures

Parameter (*n* = 10)	*C*_max_ (µg/ml)	AUC (h.mg/L)		Filter efficiency^a^	Filter efficiency at time *T*_x_ (%)^b^			
		Pre-filter	Post-filter	Overall	*T*_10_^c^	*T*_end infusion_^c^	*T*_end washout_^c^	Mean
Mean	1.13	4.29	0.57	86.0	95.4	85.9	77.5	86.3
SEM	0.13	0.28	0.0	2.5	2.1	3.6	8.1	3.7
Median	1.15	4.55	0.49	87.2	100	86.3	84.4	86.2
Minimum	0.50	2.20	0.23	71.1	82.7	63.6	30.0	68.2
Maximum	1.80	5.20	1.30	95.5	100	100	100	100
Range	1.30	3.00	1.07	24.4	17.2	36.4	70	31.8

*SEM* standard error of the mean

^a^[(AUC_pre-filter_ − AUC_post-filter_)/AUC_pre-filter_] × 100

^b^[(Pre-filter concentration) − (post-filter concentration)/(pre-filter concentration)] at time *T*_x_

^c^t10 versus t30: *p* = 0.013; t30 versus t60: *p* = 0.290; t10 versus t60: *p* = 0.051. (*p* for significance is *p* < 0.017)

**Fig. 3:**
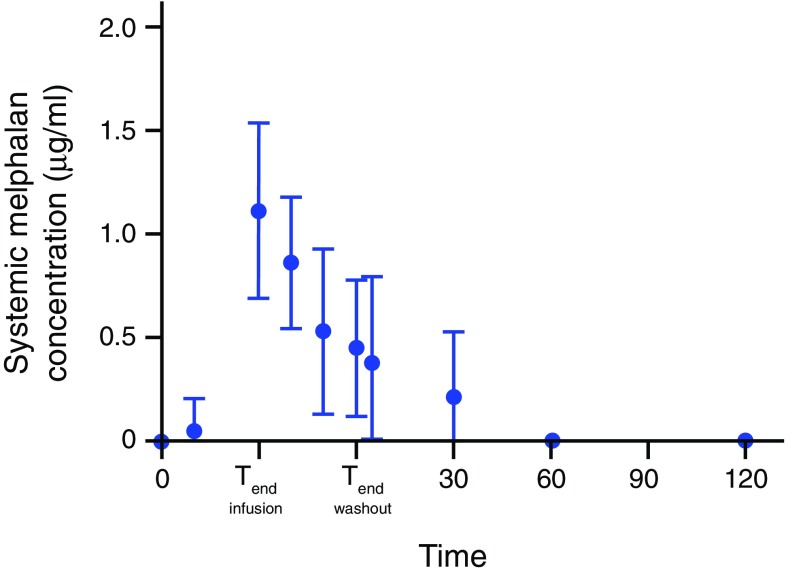
Mean systemic concentration of melphalan of all patients over time. A mean concentration of systemic melphalan was calculated at different time points; for all ten procedures, the *bars* indicate the standard deviation (SD). The *horizontal dotted line* at 0.5 µg/ml indicates the detection limit of melphalan in plasma

Figure 3 displays the mean plasma concentration of melphalan of all patients during the PHP procedure. The systemic concentration increases rapidly during the infusion period. The mean peak melphalan plasma (*C*_max_) was 1.1 µg/ml (range 0.5–1.8 µg/ml). In the majority of the procedures (67%), *C*_max_ occurred at *T*_end infusion_. The melphalan plasma concentration decreased rapidly after cessation of infusion and was undetectable in the blood samples in all patients 2 h after the start of the infusion.

### Safety of PHP

Procedure-related adverse events in all 10 PHP procedures are summarised in Table 4. This excludes peri-procedural transient hypotension, which was seen and managed successfully by the anaesthesiologist in all patients and did not result in any hypotension-related complications. Haematological laboratory disorders were the most common post-procedural complication. Early (<3 days) anaemia and thrombocytopenia grade III occurred after 10% of the procedures. No early grade III or IV leukopenia or neutropenia was observed, but asymptomatic early grade III (40%) or IV (10%) lymphocytopenia occurred after half of the perfusions. Late haematological complications, indicative of bone marrow depression, were observed in the majority of patients in our study. Late grade III/IV leukopenia, neutropenia, and thrombocytopenia were observed after 80.0, 80.0, and 40.0% of perfusions, respectively. After the first two procedures, pancytopenia occurred: the first patient was asymptomatic, but the second patient was admitted to the IC because of a bacterial pharyngitis. After this, the protocol was amended; during subsequent procedures, 6 mg of granulocyte colony-stimulating factor (GCSF) was administered 48 h after the treatment. Four patients received blood transfusion to correct post-procedural blood cell abnormalities. No relation was found between the occurrence of grade III/IV haematological complications and the administered melphalan dose. In all patients, haematological laboratory values had returned to baseline within 3 weeks. Mean time for blood cell count to return to normal was 8.3 days (range 1–20 days) for thrombocytes (normal laboratory value 150–400 × 10^9^/l) and 13 days (range 4–20 days) for leucocytes (normal laboratory value 40–10 × 10^9^/l).

**Table 4:** Main procedure-related adverse events by severity in all perfusions (*n* = 10), categorised as early phase (day 0–3) and late phase (day 4–6 weeks after perfusion)

CTCAE^a^	All grades (*n*)	Grade 3 (*n*)	Grade 4 (*n*)
*H*a*ematological events*			
Anaemia	9	1	–
Early	9	1	–
Late			
Thrombocytopenia	9	1	–
Early	9	–	4
Late			
Leucopenia	3	–	–
Early	8	1	7
Late			
Neutropenia	–	–	–
Early	8	–	8
Late^b^			
Lymphocytopenia			
Early	8	4	1
Late^b^	9	6	1
*Hepatic events*			
Elevated AST level	5	–	–
Early	3	–	–
Late^b^			
Elevated ALT level			
Early	3	–	–
Late^b^	2	–	–
Elevated serum bilirubin level			
Early	1	–	–
Late^b^	2	–	–
*Other*			
Fever	2	–	–
Thromboembolic event^c^	1	1	–
Post-procedural haemorrhage^d^	2	–	–
Pharyngitis^e^	1	1	–
Alopecia	1	–	–
Nausea	2	–	–
Oedema limbs^f^	1	–	–

*CTCAE* common terminology criteria for adverse events, *AST* aspartate aminotransferase, *ALT* alanine aminotransferase

^a^Grades of adverse events were defined according to CTCAE (version 4.0)

^b^Not determined in 1 perfusion

^c^Pulmonary emboli (PE) was diagnosed in one patient 17 days after PHP. Symptoms resolved in after treatment with low molecular weight heparin

^d^Bleeding from puncture site groin, managed conservatively

^e^Sepsis based on bacterial pharyngitis for which intravenous antibiotics and immunoglobulins were given, followed by aspiration of retropharyngeal abscess

^f^As a result of administration of intravenous fluid during procedure

### Efficacy of PHP

Although response and survival rates were not the primary end points of this pharmacological study, all patients were assessable for response evaluation. The results are displayed in Table 1. A partial response was achieved in all patients with ocular melanoma liver metastases (*n* = 4). The mean TTP in these patients was 15.5 months (range 9–28 months). In the patients with CRC metastases (*n* = 3), partial response was achieved in one patient (33.3%) and the mean TTP of this patient was 4.3 months (range 1–5 months).

## Discussion

In our study, we demonstrated an overall mean filter efficiency of 86.0% in patients undergoing PHP with the GEN 2 filter. The efficacy of this filter compares favourably to that of first-generation PHP filters. As mentioned in the introduction, the mean filter extraction rate of the first-generation filter (Hemosorba; Asahi Medical, Tokyo, Japan) was found to be 77% in a phase I study [9]. Apart from a better filter efficiency, also a more consistent performance of the GEN 2 filter was observed. The filter extraction rate varied from 71.1% to 95.5%, whereas a considerably wider range has been reported with the Hemosorba filter (range 58.2–94.7%). The mean filtration rate in our study was lower than that obtained in in vivo, pre-clinical studies. In a study including 6 pigs treated with PHP with the first-generation filter, the filter extraction rate was 99% [10]. In our study, the mean efficiency dropped from 95.4% at *T*_10_ to 77.5% at *T*_end infusion_, although the difference did not reach statistical significance. Pre-clinical studies have also shown that the filter efficiency decreases during the perfusion [10]. We hypothesise that the filter is more saturated at the end of the procedure. Based on this study finding, we recommend shortening the time that a patient is on the extracorporeal filtration system. This requires optimal coordination between members of the team performing the procedure and timely ordering of melphalan, as the short half-life of the drug mandates preparation shortly before the start of infusion. Furthermore, infusion time can be shortened by coil embolisation of variant hepatic arteries during the pre-procedural angiography. By this so-called consolidation of hepatic arterial inflow, the locations of infusion can be reduced and thus the need for repositioning of the catheter during the procedure. This strategy has been well established in the treatment for liver tumours with radioembolisation [12] [13].

The low percentage of early grade III/IV anaemia, leukopenia, neutropenia, and thrombocytopenia indicates that the modified activated carbon of the GEN 2 filter does not cause significant haemolysis. After half of the perfusions, early grade III/IV lymphocytopenia occurred. As decreases in number were much less frequent for other blood cells, the observed early lymphocytopenia may also be related to causes other than haemolysis by the filter. Factors such as pre-procedural fasting, peri-procedural stress, or administration of corticosteroids and fluids may play in role in causing lymphocytopenia. Late haematological complications, indicative of bone marrow depression related to systemic exposure to melphalan, were observed in the majority of patients in our study. The rates of bone marrow depression in our study are comparable to those reported after PHP with the first-generation filter [8]. Our study findings thus indicate that the improved filtration rate of the GEN 2 filter does not translate to lower rates of grade III/IV haematological complications. It is important to note though that grading of leukopenia, neutropenia, and thrombocytopenia according to CTCAE v4.03 is based on laboratory investigations, not on symptoms. Furthermore, in all patients haematological disorders were transient. There has been some speculation over the cause of systemic exposure to melphalan in patients undergoing PHP. It has been suggested that systemic toxicity may be related to causes other than incomplete filtration by the hemofiltration system [8].

In a small prospective study by Savier et al. [2], four patients underwent surgical isolated liver perfusion followed by one or two consecutive percutaneous liver perfusions. For the percutaneous procedures, a closed circuit was created using thread occlusion of the hepatic artery and portal vein occlusion with a transhepatic occlusion balloon. Blood returning from the hepatic veins was pumped into the hepatic artery, and no hemofiltration system was used. In all percutaneous liver perfusions, leakage of melphalan was seen and grade III or IV neutropenia occurred after two-thirds of the procedures. In the surgical procedures, systemic levels of melphalan were almost undetectable and no grade III/IV haematological complications occurred. The authors postulated that leakage may occur alongside the balloons or though veins around the common bile duct or the diaphragmatic veins. In our study, systemic exposure to melphalan may have been a caused by either incomplete filtration and/or leakage due to incomplete isolation of the hepatic circulation. Unfortunately, we were unable to differentiate between these two different causes of systemic exposure to melphalan.

Clearly, the toxicity of PHP with melphalan has to be balanced against the potential benefits. To date, there are limited treatment options for patients with metastatic ocular melanoma.

No standard systemic therapy is available, and chemotherapy, immunotherapy, or targeted therapies have not yet been able to show improved survival [14]. Radioembolisation and transarterial chemoembolisation are effective locoregional therapies for patients with primary and secondary liver tumours, but the results in patients with liver metastases from ocular melanoma have only been described in retrospective, small cohort studies [15, 16]. The superiority of PHP with melphalan over best alternative care (BAC) has been demonstrated in a multicentre RCT including 93 patients with unresectable hepatic metastases from either ocular (*n* = 83) or cutaneous (*n* = 10) melanoma [1]. The hepatic progression-free survival (hPFS) and overall progression-free survival (oPFS) in the PHP group were 7.0 and 5.4 months, respectively, compared to 1.6 and 1.6 months, respectively, for the BAC group (*p* < 0.0001). Given the potential benefit, we consider the safety profile of PHP to be acceptable in patients with hepatic metastases from ocular melanoma and PHP should therefore be considered as a first-line therapy for these patients. For patients with colorectal cancer metastases, several other treatment options are available, such as chemotherapy, radioembolisation, or targeted therapy. Therefore, the place of PHP as treatment option for these patients has yet to be determined.

The small sample size is the most important limitation of our study. Another limitation is related to the difficulties of melphalan analysis, which precluded immediate assessment of melphalan levels during the procedure and only allowed detection of melphalan above a threshold of 0.5 µg/ml. The inability to detect melphalan levels below 0.5 µg/ml may have led to overestimation of the filter efficiency at the different time points. Yet, this limitation had little influence on determination of the overall filter efficiency as this was measured as area under the curve using the trapezoid method.

In conclusion, our study demonstrates that the filtration rate of the GEN 2 hemofiltration system performs better than the first-generation filtration system. The filter efficiency decreases during the PHP procedure. Despite the improved filtration rate, haematological laboratory disorders grade III/IV are common, but these are transient and usually asymptomatic.
