# Erratum to: Prospective Clinical and Pharmacological Evaluation of the Delcath System’s Second-Generation (GEN2) Hemofiltration System in Patients Undergoing Percutaneous Hepatic Perfusion with Melphalan

**DOI:** 10.1007/s00270-017-1766-2

**Published:** 2017-08-23

**Authors:** Eleonora M. de Leede, Mark C. Burgmans, T. Susanna Meijer, Christian H. Martini, Fred G. J. Tijl, Jaap Vuyk, Arian R. van Erkel, Cornelis J. H. van der Velde, Ellen Kapiteijn, Alexander L. Vahrmeijer

**Affiliations:** 1 Department of Surgery, Leiden University Medical Centre, Albinusdreef 2, 2300 RC Leiden, The Netherlands; 2 Department of Radiology, Leiden University Medical Centre, Albinusdreef 2, 2300 RC Leiden, The Netherlands; 3 Department of Anesthesiology, Leiden University Medical Centre, Albinusdreef 2, 2300 RC Leiden, The Netherlands; 4 Department of Cardio-Thoracic Surgery, Leiden University Medical Centre, Albinusdreef 2, 2300 RC Leiden, The Netherlands; 5 Department of Medical Oncology, Leiden University Medical Centre, Albinusdreef 2, 2300 RC Leiden, The Netherlands

## Erratum to: Cardiovasc Intervent Radiol (2017) 40(8):1196–1205 DOI 10.1007/s00270-017-1630-4

The article [Prospective Clinical and Pharmacological Evaluation of the Delcath System’s Second-Generation (GEN2) Hemofiltration System in Patients Undergoing Percutaneous Hepatic Perfusion with Melphalan], written by Eleonora M. de Leede et al., was originally published Online First without open access. After publication in volume 40, issue 8, pages 1196–1205 it was discovered that this article should have been published open access (with the APC having been paid by the corresponding author’s institution as part of their Compact agreement). The incorrect license was assigned to this paper due to a technical error. Therefore, the copyright of the article has been changed to © The Author(s) 2017 and the article is forthwith distributed under the terms of the Creative Commons Attribution. The original article was corrected.

